# Study on Lateral-Load Resisting Mechanism and Capacities of Steel Frame Infilled with Composite Plate Shear Wall Under Cyclic Loading

**DOI:** 10.3390/ma18071677

**Published:** 2025-04-06

**Authors:** Hui Li, Yi Qi, Tongyang Kang, Huafei Wang

**Affiliations:** 1 School of Civil Engineering, Suzhou University of Science and Technology, Suzhou 215011, China; 2213031086@post.usts.edu.cn (H.L.); 2211032007@post.usts.edu.cn (T.K.); 2 School of Intelligent Manufacturing and Smart Transportation, Suzhou City University, Suzhou 215104, China; wanghf@szcu.edu.cn

**Keywords:** frame-infill shear wall, composite shear wall, shear mechanism, lateral resistance, finite element method, mechanics-based model

## Abstract

Steel frame infilled with composite plate shear wall (SF-CPSW) is an effective structure for lateral-load resisting. In the structural design, the vertical loads are primarily carried by the boundary SF, while the horizontal loads are expected to be totally carried by CPSW. CPSW incorporates the steel web and the concrete encasements. For the CPSW bays, the boundary SF also inevitably withstands the lateral-loads due to the coordinated deformations between boundary SF and CPSW. The available researches, however, have not given a certain shear force assignment between the boundary SF and CPSW. Furthermore, their interactions under the cyclic lateral-loading are unclear. This paper conducted a study on the load-resisting mechanism of SF-CPSW by a structural model test and finite element analyses. The deformation pattern, failure mode, internal forces, and interactions of structural members were investigated. The effects of steel web and concrete thicknesses, cross-sections of boundary SF, and axial compression ratio on the lateral-load resistance of SF-CPSW were assessed. The results indicated that the interactions of CPSW and boundary SF caused significant normal stresses at the corners of CPSW, reducing the shear strength of steel web. However, the concrete encasements and boundary SF compensate it and mutually improved the stiffness and ductility. According to the analysis results, the formulas of the lateral stiffness and strengths of SF-CPSW were proposed for its seismic design.

## 1. Introduction

Steel frame infilled with composite plate shear wall (SF-CPSW) is an effective lateral-load resisting structure, which developed from the steel plate shear wall (SPSW) [1]. The main components of composite plate shear wall (CPSW) are steel web, reinforced concrete, and connectors. Composite plate shear wall/concrete-encased (C-PSW/CE) is one of the commonly used types, which is the reinforced concrete encasements placed on one or both sides of the infill steel web. The concrete encasements can restrain the out-of-plane buckling of the infill steel web [2]. As a result, the infill steel web maintains in-plane shearing. Due to its desirable performance, CPSW has been widely investigated in its lateral load resistance by employing different methods. For example, the shear capacity and ultimate strength of CPSW would not increase when the concrete thickness beyond a specific value [3]. Moreover, the layout of the shear connectors also affects the deformation of CPSW. The ductility of CPSW could improve when the shear studs spacing increased up to a specific value [4,5,6]. Based on these, Qi, Gu, Wang et al. proposed the requirement on the concrete encasements and internal forces of studs [7,8,9,10].

In addition, some scholars have made some innovations to CPSW, for example, using different size, location, and shape of openings [11] to investigate the lateral-load resistance and equate different types of openings by circular openings [12,13], using lightweight [14] and high-performance fiber-reinforced concrete [15] to enhance the strength and ductility. To achieve the damage-free behavior and high ductility, the rubber-coated uplift-restrained stud was put forward [16]. Furthermore, the modular prefabricated composite shear panel was proposed [17,18].

The above researches are evident that CPSW exhibited high lateral load resistance and deformation capability under the cyclic loading. In SF-CPSW structures, the previous studies suggested that the vertical loads were primarily carried by the boundary steel frame (SF), while the horizontal loads were expected to be totally carried by CPSW. However, the contributions of boundary SF to the lateral resistance of SF-CPSW were neglected. In service, the boundary SF also withstands partial lateral load.

Based on the previous studies, some researches demonstrated that the steel frame was not just as boundary conditions. The boundary SF also carries lateral force in the cyclic lateral loads. Dey and Bhowmick proposed a basic period calculation formula for C-PSW/CE under different seismic conditions [19]. Frhbakhshtooli pointed out that the boundary column of C-PSWs resisted a large part of the inter-layer shear. The additional strength and stiffness contribution of the column would provide a valuable backup for the infill steel web [20]. And the ductility and strength-modification factors specified in AISC341-16 were not applicable for C-PSW/CE [21]. Shafaei, Farahbod, and Ayazi researched that as the drift ratio increased, the shear resistance of the steel web decreased due to the shear yield. Afterwards, the shear resistance contribution of the steel frame gradually increased [22]. Lei, Qi, and Gu determined that the energy dissipation of SF-CPSW was primarily due to shear transfer and bending constraint effects, which resulted in plastic shear in the infill steel web and the beam web [23].

## 2. Innovation and Methodology

As yet, the numerous studies demonstrated that SF-CPSW could reach an excellent seismic performance [1] and the boundary SF also withstood partial lateral-load, rather than the vertical load [20]. Although the interactions between the boundary SF and CPSW were investigated by a few researchers [22,24], the difference of the acting forces (from CPSW to SF) among the steel web and concrete encasement was neglected and their distributions were simplified. Furthermore, the contributions of boundary SF to the lateral load resistance were proposed without considering the interactions between boundary SF and CPSW. As a result, how the infill steel web achieves shear yielding and the contribution of boundary SF to the ductility are unknown. Accordingly, the load-resisting mechanism of SF-CPSW is still unclear. In this paper, the load-carrying mechanisms of infill steel web, concrete encasements, and the boundary SF, and their interactions and deformation pattern, were analyzed and revealed. Grounded on these, the shear stiffness and strengths of CPSW combining with the boundary SF were proposed.

This paper was divided into three parts involving the test, finite element analysis, and theoretical analyses to conduct the aforementioned investigation. Through the test, the failure modes and mechanics characteristics, including the deformations and strains of SF-CPSW, could be obtained by the measurements of *V*-*U* curves and strains and the observations. A total of 81 finite element models (FEMs) were established and analyzed by ABAQUS software [25] to perform the parametric analyses and to obtain the comprehensive data, including the internal forces, interaction forces, and the stresses for the theoretical analyses and design method. Meanwhile, the finite element modeling method was verified by the test results. According to the FEM results, the load-resisting mechanism of SF-CPSW was deduced and the associated formulas were proposed by theoretical analyses.

## 3. Experimental Program

To obtain the failure modes and mechanics characteristics of SF-CPSW under lateral loading, a 1:3 scaled SF-CPSW specimen was designed and tested. This section introduced its experimental program, including specimen design and material properties, test setup, loading procedure, and instrumentation arrangements.

### 3.1. Specimen Design and Material Properties

**Figure 1:**
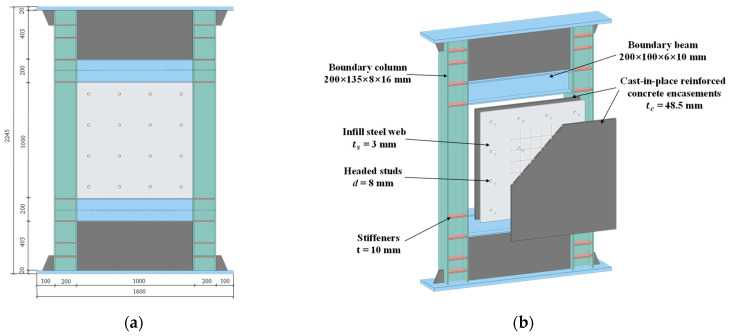
Specimens schematic. (**a**) Dimensions of specimen. (**b**) Components of specimen.

A 1:3 scaled SF-CPSW specimen was designed by following the rule of “geometric and mechanical similarity” for the experimental research. The specimen was two storeys (one full and two half storeys) and consisted of infill steel web, cast-in-place reinforced concrete encasements, rebars, headed studs, and the boundary SF, as shown in Figure 1. The height and span of SF-CPSW were 2245 and 1600 mm, respectively, whereas the heights of full storey and half storeys were 1000 and 403 mm, respectively. The thickness of CPSW was 100 mm, while the thickness of infill steel web was 3 mm. The concrete encasements on both sides were equal to 48.5 mm, which met the thickness demand for concrete encasements [8]. The reinforcement ratios of concrete encasements met the minimum value of 0.25% as stipulated by [26]. Headed studs with 8 mm diameter were used to connect the concrete encasements and infill steel web, meeting the requirements of the headed stud demand proposed in [9,27] and requirements per [28]. The beams and columns of boundary steel frame were H-shaped cross-sections with the dimensions of 200 × 100 × 6 × 10 mm and 200 × 135 × 8 × 16 mm, respectively.

The material properties of different steel thickness *t*_0_ were obtained from three tensile coupons, according to the GB/T228-2021 [29]. The axial force and corresponding deformation of the tensile coupons were recorded by the data acquisition system. The relative longitudinal displacement within the gauge length was measured using an extensometer. Based on these measurements, the averages of the Young’s modulus *E*_s_, yield strength *f*_y_, and ultimate strength *f*_u_ were tested, as listed in Table 1. The elongation percentages *δ* were calculated by dividing the residual elongation of the fractured gauge length by the original gauge length.

Due to the thin section of concrete encasements, the ready-mix concrete with a maximum coarse aggregate size of 16 mm was selected. During the concrete pouring, three concrete cubes (150 × 150 × 150 mm) were made and maintained under the same conditions as the test specimen to measure their compressive strength. The characteristic compressive strength of concrete cube (*f*_cu,k_) was designed as 20 MPa. The average of measured ones was 22.3 MPa, which satisfied the stipulation for the concrete-strength grade with *f*_cu,k_ equal to 20 MPa [30].

### 3.2. Test Setup and Loading Procedure

**Figure 2:**
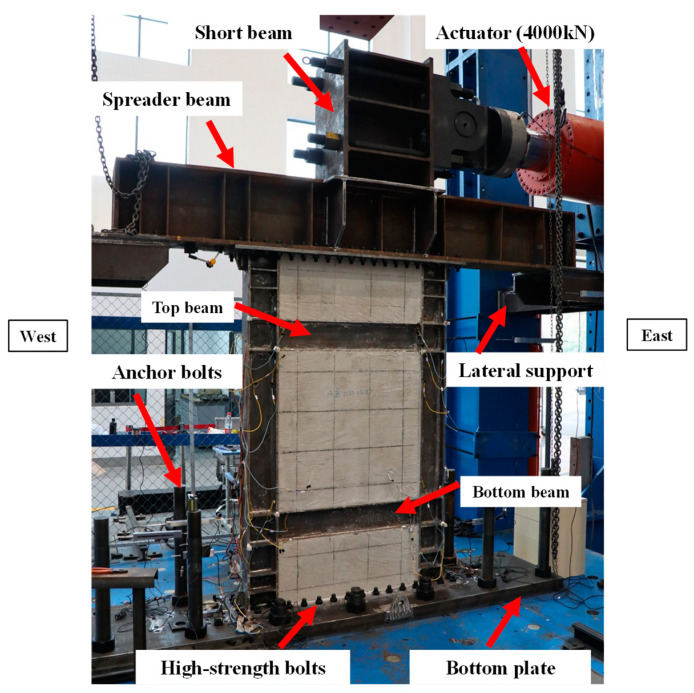
Test setup and boundary conditions.

The test setup was shown in Figure 2. The main components were horizontal actuator, loading beam, bottom plate, and lateral supports. The actuator could provide a maximum load of 4000 kN and a maximum displacement of ±250 mm. During the test, the actuator horizontally applied the quasi-static cyclic loading to the loading beam, thereby transmitting lateral loads to the specimen. The whole loading beam was composed of a short beam and a spreader beam; both ends of the short beam were connected with the actuator through diameter screw. The top and bottom of the specimen were respectively connected to the loading beam and bottom plate by the high-strength bolts, and the bottom plate was anchored to the foundation.

To prevent the specimen from undergoing out-of-plane displacement during the cyclic loading, both sides of the loading beam installed the lateral supports. These lateral supports were connected to both ends of the lower flanges of the loading beam by high-strength bolts. The outer surfaces of these supports were connected to the acrylic plates, approximately with a 5 mm gap to the inside of the loading device columns.

The loading of the specimen was displacement controlled. Before the infill steel web yielded, the cycle number was one; after the yielding, the cycle number became three. When the strength deterioration of the specimen was over 15%, the loading process came to an end.

### 3.3. Instrumentation Arrangements

**Figure 3:**
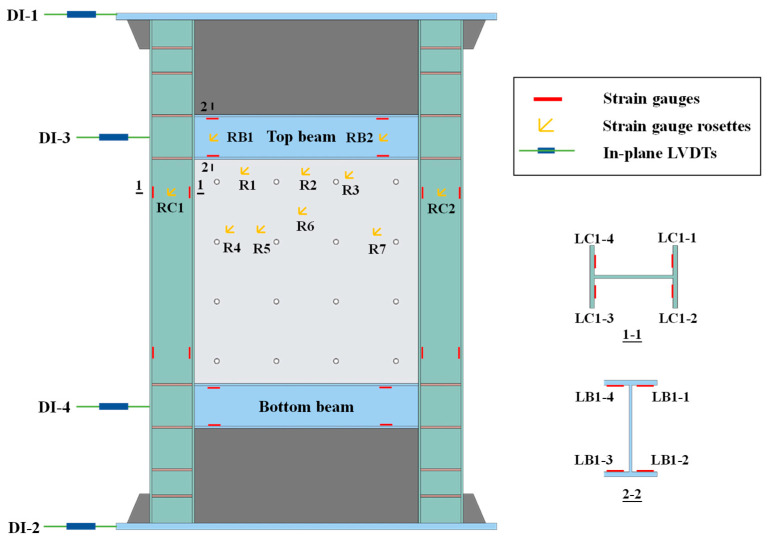
Instrumentation arrangements.

The responses of the specimen were measured through the in-plane linear variable differential transformers (LVDTs) and electrical resistance strain gauges, as shown in Figure 3. Four LVDTs located in the in-plane of the specimen were set up. The LVDTs were represented by DI-i, where i was the number. DI-1 and DI-2 were placed along the center axes of the top and bottom plates, and DI-3 and DI-4 were placed along the top beam and bottom beam, respectively, to monitor the horizontal displacements. The triaxial strain gauge rosettes were installed on the steel web and the webs of beams and columns to monitor the shear deformation, labeled as R*i*, RB*i*, RC*i* (where B represented the beam and C was for the column). Meanwhile, four uniaxial strain gauges were symmetrically arranged at flanges of beams and columns to measure the strains resulting from bending, labeled as LB*i* and LC*i*.

## 4. Experimental Results

The failure modes and mechanics characteristics of the specimen under the lateral loading were analyzed through load-displacement development and observations of the test. Additionally, the analyses on the strain developments and distributions of the specimen were conducted, based on the data collected from the instrumentations.

### 4.1. Load-Displacement Development

**Figure 4:**
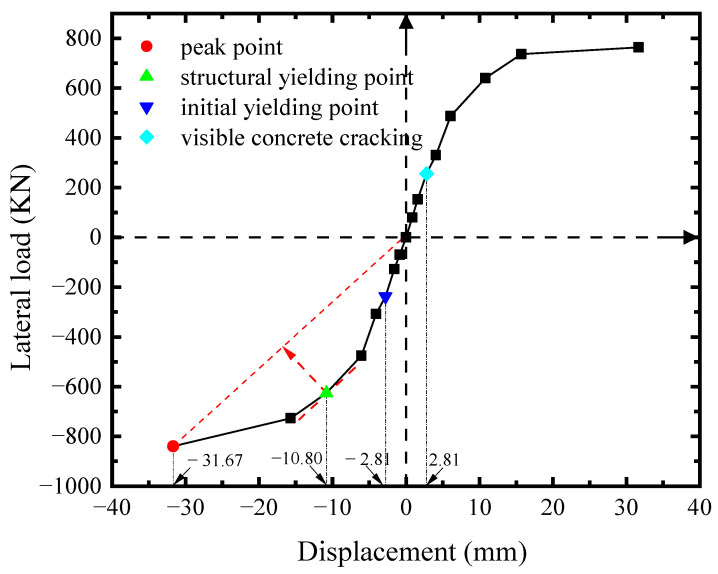
*V*-*U* response of specimen.

The skeleton curve of lateral load-displacement (*V*-*U*) of the specimen was analyzed via the significant points, characterized by the experimental phenomena and instrument readings, as shown in Figure 4. The loading direction was negative from west to east. The skeleton curve remained a linear response until *U* = −2.81 mm. At this point, the infill steel web initially yielded in shear. After that, the specimen entered the elastoplastic phase, and its shear capacity reduced correspondingly. At the same loading level of *U* = +2.81 mm, the visible cracks of concrete encasements emerged. As the plasticity of the steel web developed, *V*-*U* curve reached the farthest point along the line of original point to the peak point at *U* = −10.80 mm. This point was identified as the structural yield point per [31], and it was characterized by the initially yielding of boundary column. When *V* reached the maximum of −840 kN at *U* = −31.67 mm (the peak point), the plastic out-of-plane buckling of boundary columns occurred, and *V* drastically drop at the next loading level. Considering the safety, the test had to be terminated at the same time. Obviously, the loading location added the overturning moment to the specimen, which triggered the premature plastic buckling of columns.

### 4.2. Observations and Failures

**Figure 5:**
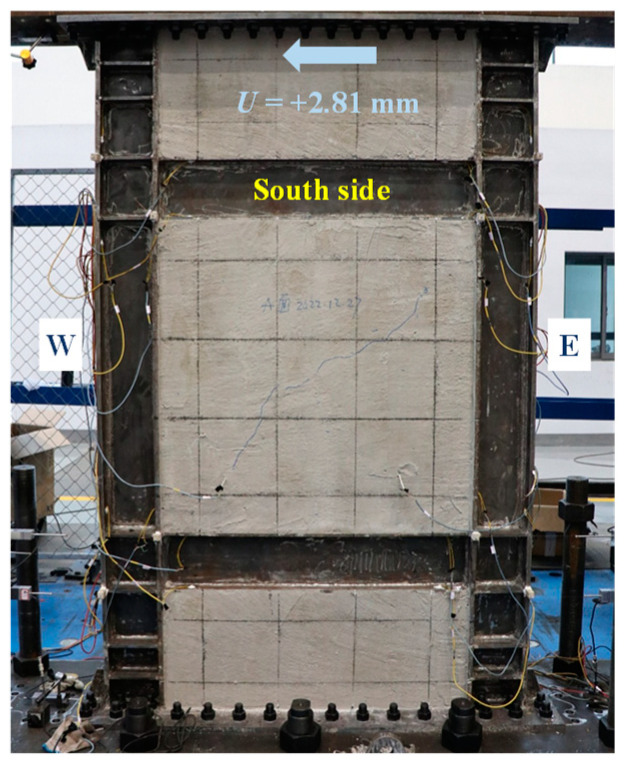
Onset of concrete cracking.

**Figure 6:**
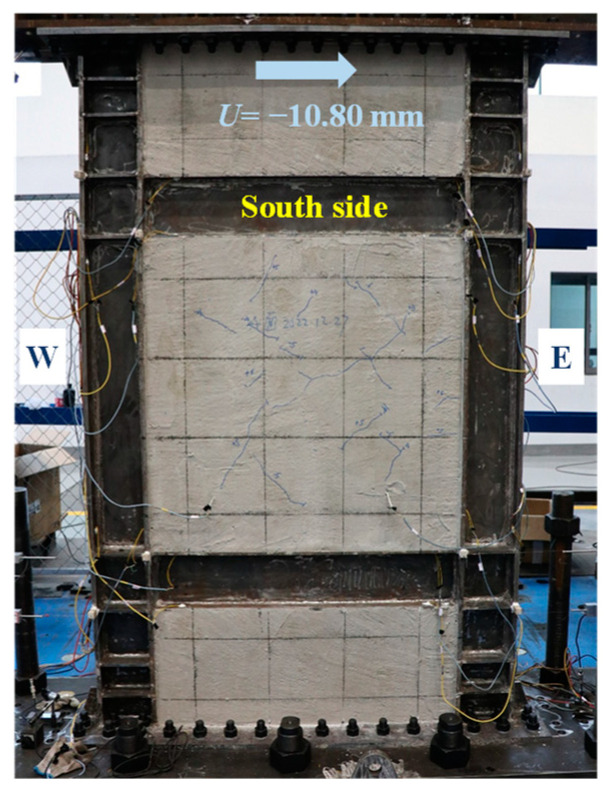
Flexure deformations of the column.

**Figure 7:**
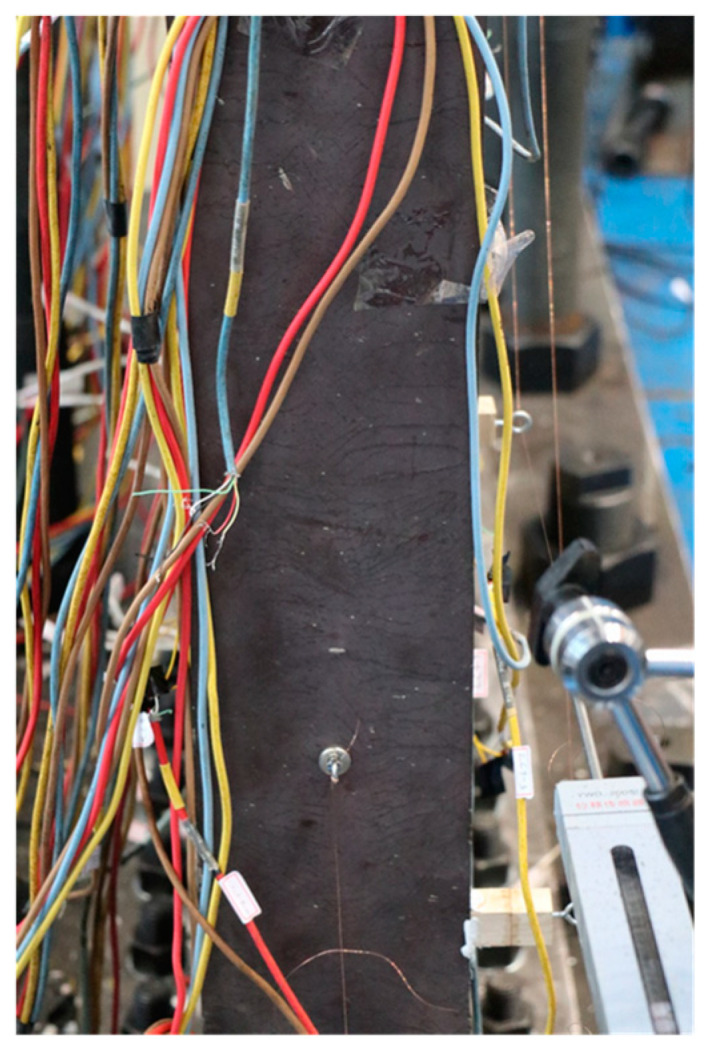
Tiny cracks of the column flange.

**Figure 8:**
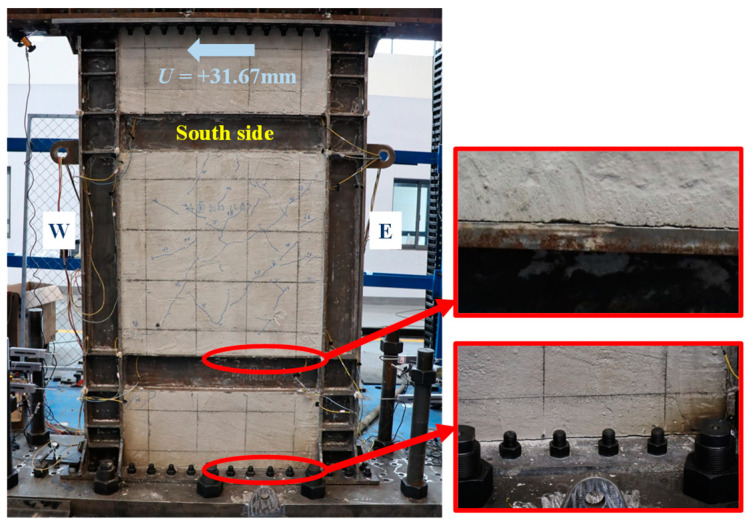
Separations of concrete encasement.

**Figure 9:**
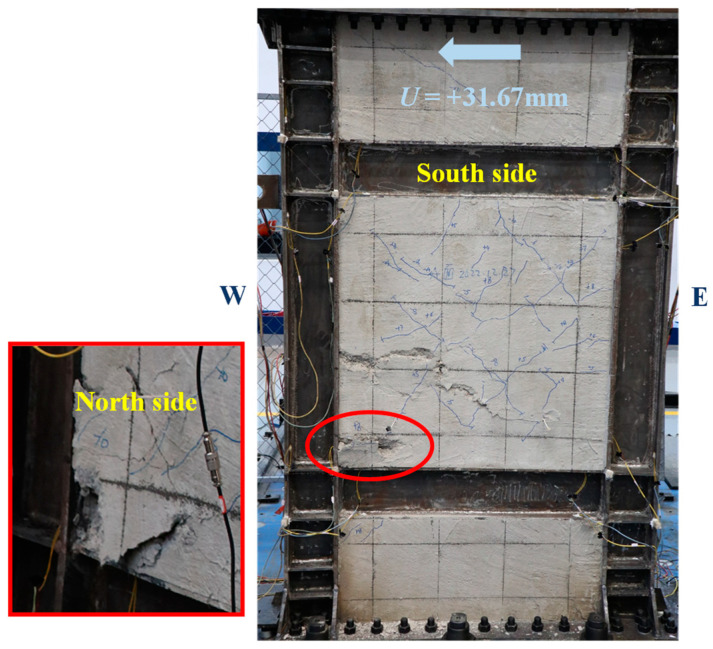
Onset of concrete crushing.

**Figure 10:**
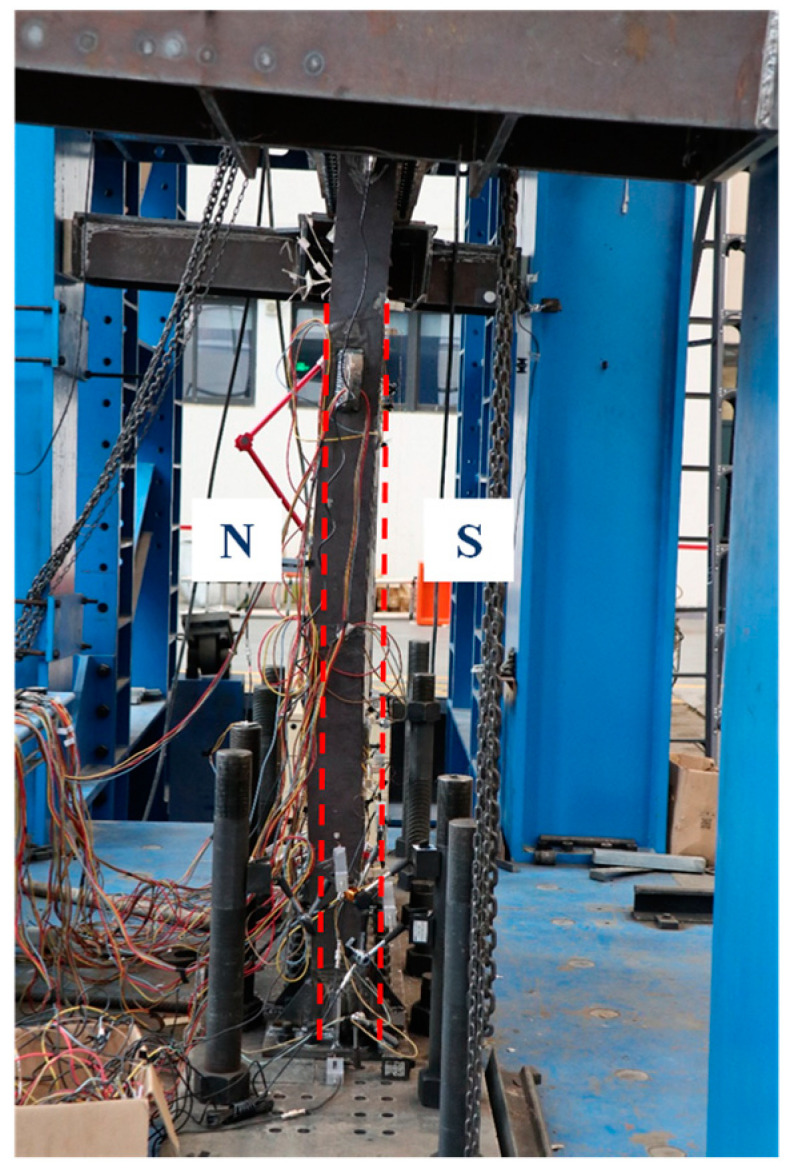
Twisting tendency of SF-CPSW.

Figure 5, Figure 6, Figure 7, Figure 8, Figure 9 and Figure 10 illustrated the observations of the test. As shown in Figure 5, the initially visible concrete cracks appeared at *U* = +2.81 mm and approximately developed along 45°. It implied that concrete encasements were mainly subjected to the shear deformation. At the structural yield point (*U* = −10.80 mm), due to the constraint from CPSW, the overall deformation of boundary SF was dominated by the shear. At the column-beam joints, however, some flexure deformations were developed, as shown in Figure 6. At the same loading level, more cracks emerged and crossed under the cyclic loading, coinciding with the column yielding. Accordingly, tiny cracks extended on the surface of the column flange, as shown in Figure 7. At the peak point of *V*-*θ* (*U* = +31.67 mm), some separations between beam flange or bottom plate and concrete encasements formed, as shown in Figure 8, indicating the increment of the relative deformation between CPSW and boundary SF. Meanwhile, the west column plastically buckled to the north side, leading to the concrete crushing on the south side, as illustrated in Figure 9. On the north side, the concrete encasements fully spalled and exposed the rebars at the corner. Such out-of-plane buckling transmitted a twisting tendency in SF-CPSW, as shown in Figure 10, causing a significant deterioration of load-carrying capacity of SF-CPSW. Thus, the test was terminated. Although the failure mode might not be desirable, the strains responses of structural members before the peak load was adequately valuable to this research.

### 4.3. Strain Developments and Distributions

**Figure 11:**
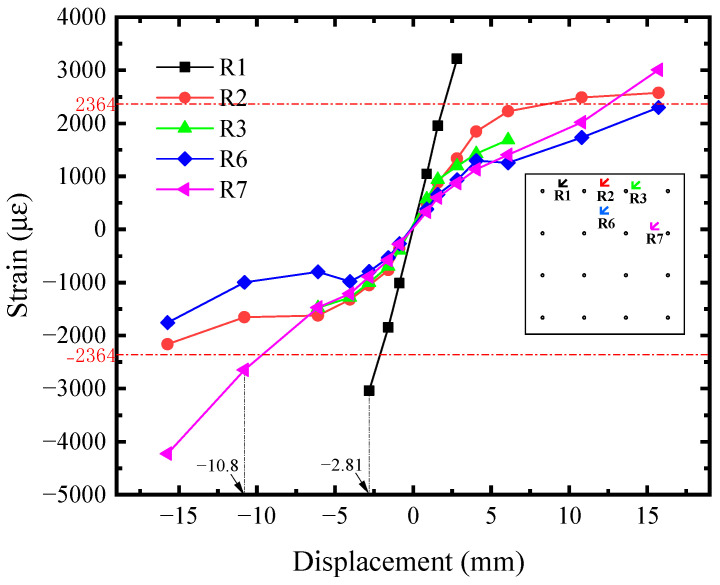
Shear strain of infill steel web.

**Figure 12:**
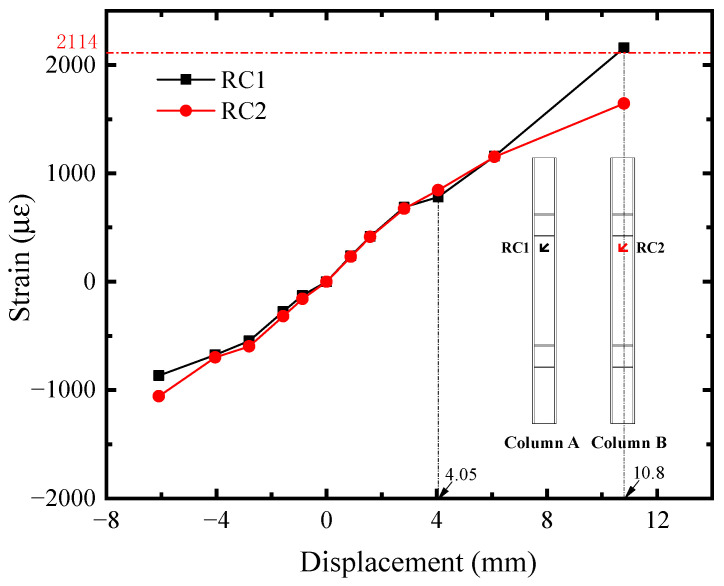
Mises strain of column.

**Figure 13:**
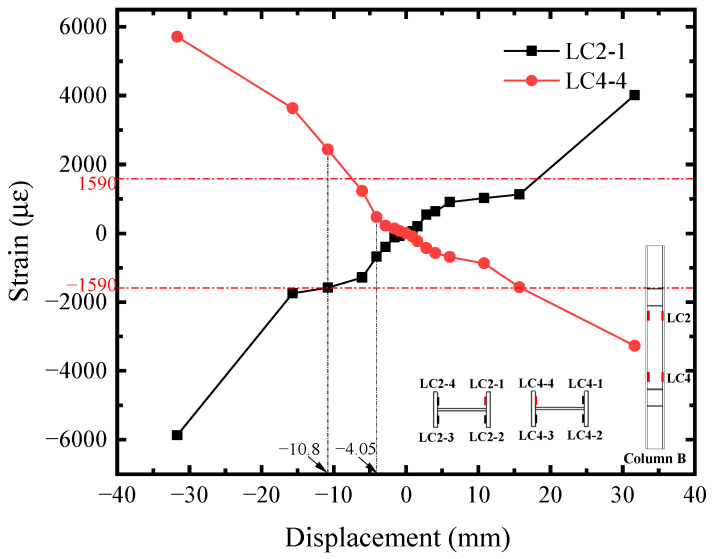
Axial strain of column.

**Figure 14:**
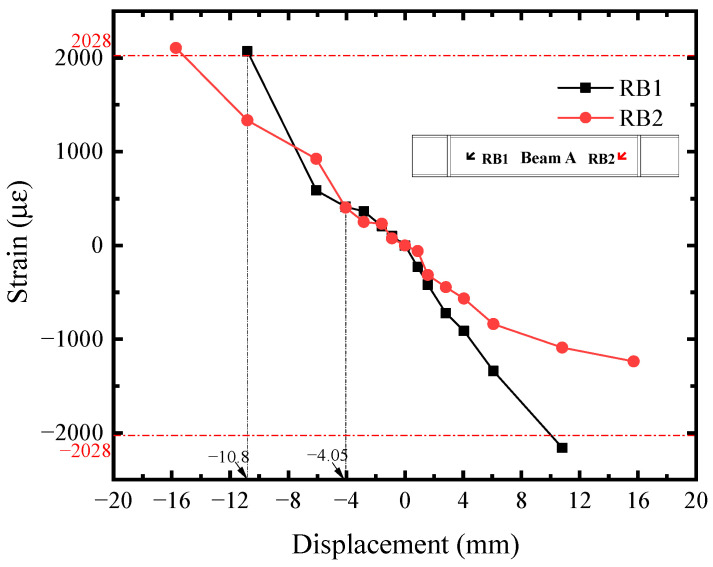
Shear strain of beam.

The shear strain-displacement (*R*_i_-*U*) responses of infill steel web were shown in Figure 11. Its corner part yielded first. Seeing the R1 curve, it reached the shear yield strain (2364 *με*) at *U* = −2.81 mm, implying the initial yielding of infill steel web. It was consistent with the initial yielding point in Figure 4. For the rest position, their shear strains were almost equal before *U* = −4.05 mm and lower than R1. Afterwards, the curves developed with different tendency, which was affected by the concrete cracking. R2, R6, and R7 exceeded or reached the yield strength approximately at *U* = −10.80 mm, indicating that the plasticity of infill steel web had been significantly developed at the structural yield point of SF-CPSW. Meanwhile, in the boundary SF, the yield onsets of beam web as well as the full cross-section of column ends occurred, as illustrated in Figure 12, Figure 13 and Figure 14. Figure 12 also revealed that the flexure of column ends became apparent after *U* = 4.05 mm, in accordance with the observation in Figure 6. Affected by the overturning moment from SF-CPSW, the axial force of columns was higher than the beams and speeded the fully yielding of their cross-sections. The strain developments of column flanges and webs, as shown in Figure 12 and Figure 13, confirmed it. For the boundary beams, as the constraints to their bending deformations from CPSWs were stronger than that to the columns, their initial yield was determined by shear and occurred in the webs, as shown in Figure 14.

## 5. Finite Element Modeling Method

To further analyze the mechanics characteristics of SF-CPSW, finite element analyses were conducted. This section presented the finite element method, including element types and constrains, the material properties of elements. The modeling method was verified by two test results, where one was obtained in this paper, and the parameters of 81 specimens for the finite element analyses were listed.

### 5.1. Element Types and Constraints

The finite element model (FEM) of SF-CPSW was established by ABAQUS software. The FEM methodology for SF-CPSW was introduced in earlier work by the authors [9]. To analyze the hysteretic behaviors of SF-CPSW in practice, the FEMs herein were full-scale and consisted of two storeys. Their boundary conditions were the same as the test.

**Figure 15:**
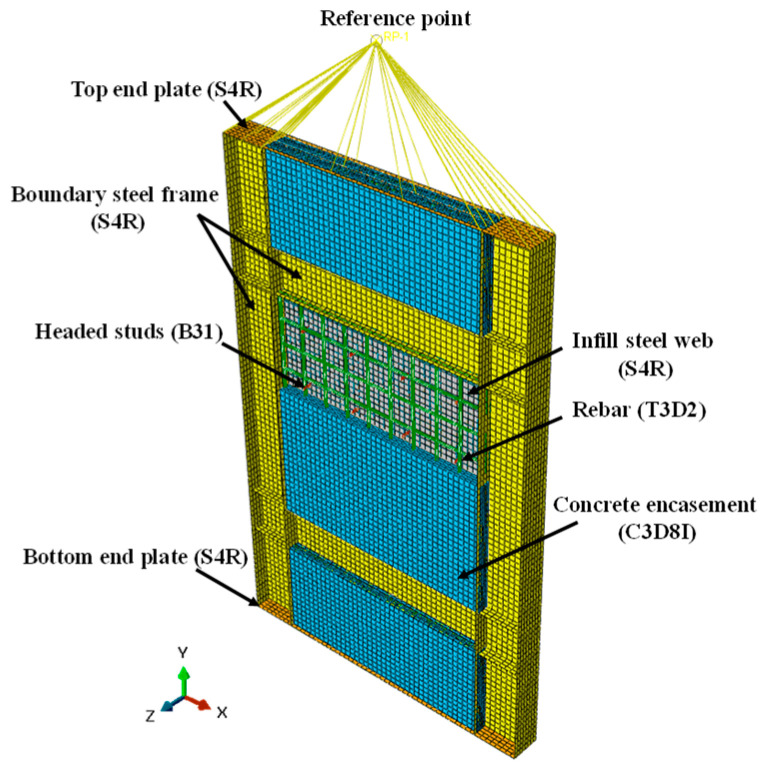
Finite element model.

As shown in Figure 15, the infill steel web, boundary SF, and end plate were built using shell elements with 4-node (S4R). The concrete encasements were simulated by incompatible mode 8-node brick elements (C3D8I). The headed studs and the rebars were built by beam elements (B31) and truss elements (T3D2), respectively.

The welds between the structural members of SF-CPSW were simplified to rigid connections, using common nodes or “tie” constraint to couple all degrees of freedom (DoF) of the elements. The common nodes constraints were employed among the element nodes of the boundary SF, infill steel web, and the headed studs. The infill steel web and the boundary SF were connected by the “tie” constraints. The interactions of the overlap surfaces on the concrete encasements, infill steel web, and flanges of the boundary frame were simulated by “contact” constraint. Its normal behavior was simulated by “hard” contact, and the tangential behavior was considered as the friction action with a coefficient of 0.6. The headed studs and rebars were embedded in the concrete encasements.

The imperfection of the infill steel web was taken as the deflection that was 1/1000 of its height. The bottom end plate of FEM was fixed to the ground. A displacement-controlled loading protocol was adopted at the coupling reference point of the top end plate, as shown in Figure 15.

### 5.2. Material Properties of Elements

The Concrete Damaged Plasticity (CDP) model in ABAQUS software version 2020 [25] was employed to simulate the material properties of concrete, where its axial tensile strength *f*_tk_ and axial compressive strength *f*_ck_ were 2.01 MPa and 20.1 MPa; the Young’s modulus was 30,000 MPa; the Poisson’s ratio was 0.2. The nonlinear behaviors of concrete were taken by the plastic parameters and the relationships of tensile damage-cracking strain and compressive damage-inelastic strain in CDP model. The specific data had been listed in previous work by the authors [9]. For the steel materials, their Young’s modulus was 206 GPa and their Poisson’s ratio was 0.3. The combine hardening model was adopted to simulate infill steel web and the boundary SF. The related parameters were taken from the test results obtained by Shi [32]. The rebars were simulated by the ideal elastoplastic constitutive model, and the nominal yield strength was 300 MPa. The headed studs that connected the infill steel web and concrete encasements utilized the bilinear kinematic hardening model with the nominal yield strength of 240 MPa and ultimate strength of 400 MPa.

### 5.3. Validation of FEM

**Figure 16:**
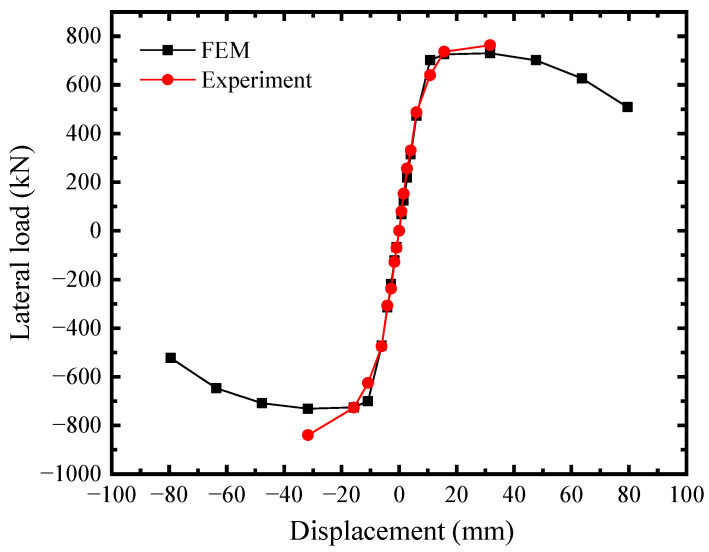
Comparison in *V*-*U* of SF-CPSW.

**Figure 17:**
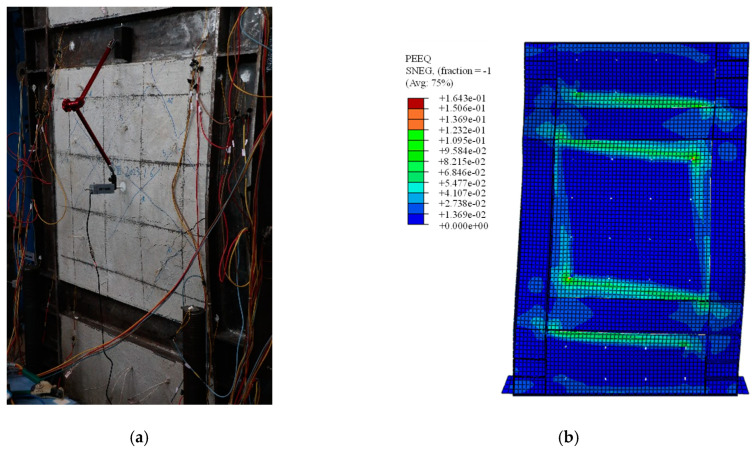
Comparison of failures of SF-CPSW. (**a**) Test specimen of SF-CPSW. (**b**) FEM of SF-CPSW.

**Figure 18:**
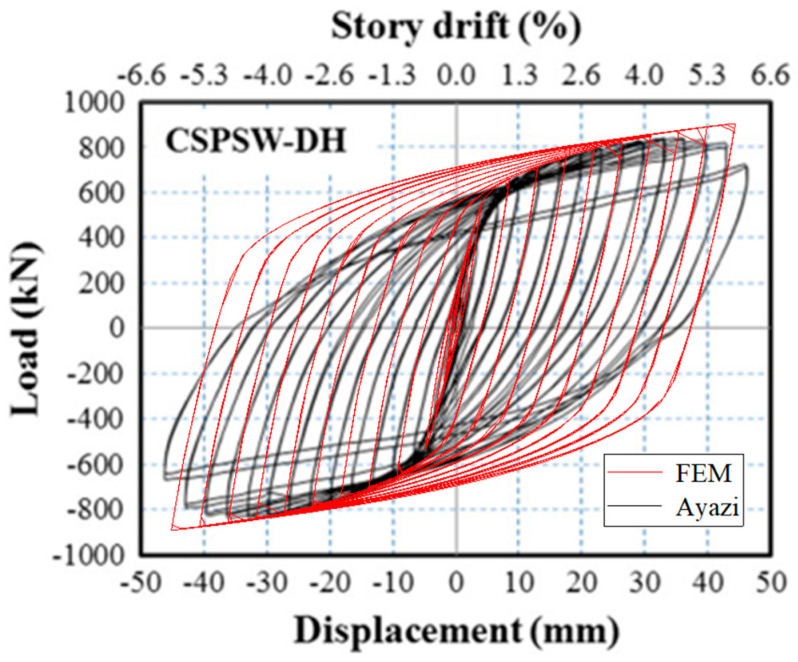
Comparison in *V*-*U* of CSPSW-DH.

**Figure 19:**
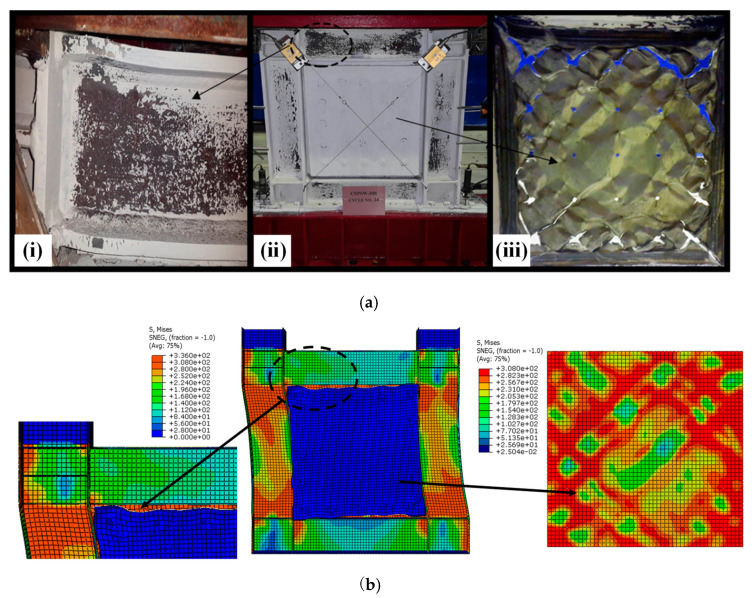
Comparison of shear behaviors of CSPSW-DH. (**a**) The failure mode of specimen of CSPSW-DH: (**i**) specimen CSPSE-DH in cycle 24; (**ii**) local buckling of the flange upper beam; (**iii**) the infill steel web at the end of the test [15]. (**b**) FEM of CSPSW-DH.

To validate the precision of FEM, the behaviors of SF-CPSW under cyclic loading were compared between the available experiments and FEM established in this paper. The boundary conditions and component materials of FEM were consistent with those of the tests. First, the test results in this paper were simulated. Figure 16 illustrated their *V*-*U* responses with significantly close developing patterns, initial stiffness, and the peak points. The peak shear forces on the positive direction of experiment and FEM were 763 kN and 730.28 kN, respectively, differing by 4.5%. After that, the test was terminated and the shear resistance of FEM gradually declined, because both of them failed by the twisting of SF-CPSW. The failure mode was compared in Figure 17. As such failure mode was unexpected, a specific imperfection with twisting modes needed to be introduced in FEM for simulation. Thus, to further validate the accuracy of FEM, another experiment study on SF-CPSW conducted by Ayazi [15] was analyzed by the proposed FEM for the validation. This specimen with one-storey and one-span of SF-CPSW was named CSPSW-DH, which used precast HPFRC encasements. The hysteretic performances of CSPSW-DH between test and FEM were similar, as shown in Figure 18. The initial stiffness of FEM matched the experiment well. The peak shear forces of the experiment and the FEM were 860.0 kN and 837.9 kN, respectively, differing by 2.7%. However, some disagreements appeared in the hysteretic curves, because the stress-strain relationships of the steel and concrete in FEM were simplified by the constitutive models employed in this paper. The failures between the experiment and FEM had a good agreement, as shown in Figure 19. Consequently, the FEM of SF-CPSW proposed in this paper could predict the load-displacement development pattern, initial stiffness, and deflection points of SF-CPSW under the cyclic loading with a reasonable accuracy.

### 5.4. FEM Specimens

In order to reveal the shear mechanism of SF-CPSW and fully evaluate its shear capacity, 81 FEM specimens were designed and listed in Table 2. Their total height and width of specimens were 7200 mm and 4200 mm, respectively, and the height and width of the middle storey were equal to 3600 mm. The beams and columns were the H-shaped cross-section with unchanged height and width of 600 × 300 mm and 600 × 400 mm, respectively. The reinforcement ratios of concrete encasements exceeded 0.25%, satisfying the requirement from AISC 341-16 [21]. The headed studs were spaced by 750 mm. The varied parameters of the FEM specimens were the infill steel web and concrete encasements thicknesses, the beam and column cross-sections, and the axial compression ratios of column. The thicknesses of infill steel web were taken as 10 mm, 12.5 mm, 15 mm, 17.5 mm, and 20 mm. The single-side thicknesses of concrete encasements were 100 mm and 150 mm. The beam and column cross-sections changed as per the webs and flanges thicknesses, as listed in Table 2. The axial compression ratios (*μ*) were selected as 0.0, 0.2, 0.4, and 0.6.

TSx-Bx-Cx-TCx-x were taken to represent the FEM specimens. For example, TS15-B2440-C3060-TC100-0.2 represented that the thickness of infill steel web was 15 mm (TS15), the web and flange thicknesses of beam were 24 mm and 40 mm (B2440), the web and flange thicknesses of column were 30 mm and 60 mm (C3060), the thickness of single-side concrete encasement was 100 mm (TC100), and 0.2 was the axial compression ratio. When the axial compression ratio was 0.0, this last item was ignored.

## 6. Finite Element Analysis on Internal Forces and Interactions of SF-CSPW

In this section, specimen TS10-B2032-C2048-TC150 was taken as an example to reveal the resistance, internal forces, and interaction mechanism of SF-CSPW under cyclic loading. Firstly, the cross-sectional division of SF-CPSW was presented to study the internal forces and the interactions of each structural element. Then, the *V*-*θ* curve, the responses, the interactions, the shear stiffness, and resistance assignments of structural elements were analyzed.

### 6.1. Cross-Sectional Division of Specimen

**Figure 20:**
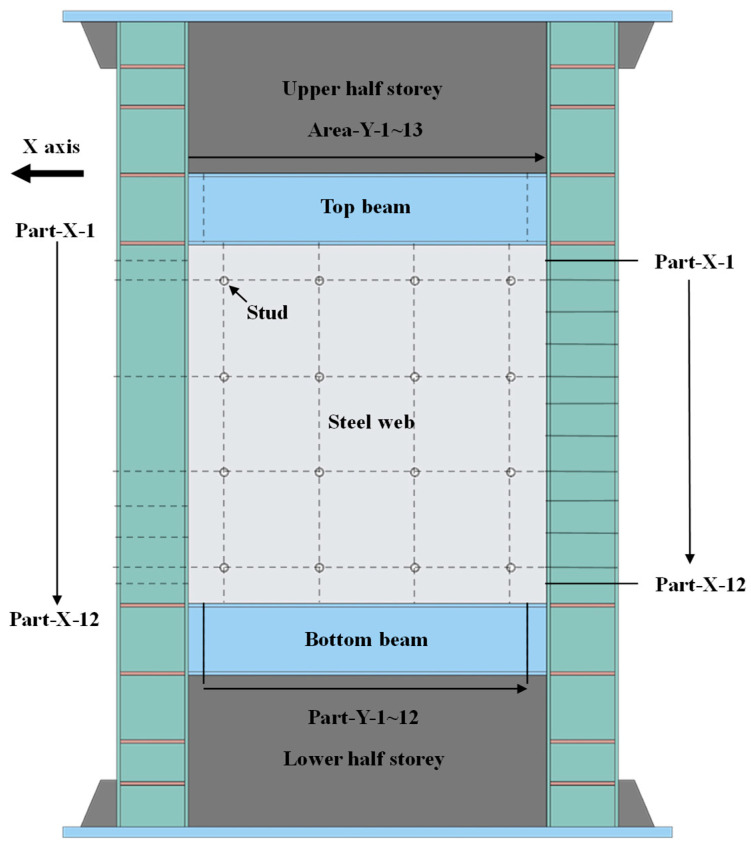
Cross-sectional division of specimen.

To study the internal forces and interactions of FEM specimens, the CPSW and boundary SF in the middle storey were divided into 12 parts and 13 areas in the horizontal (marked “X”) and vertical (marked “Y”) direction, respectively, shown as in Figure 20. The cross-sections of infill steel web, frame beam, column, and concrete encasements were denoted by SW, COL, BEAM, and CON, respectively.

### 6.2. Shear Force-Drift Ratio Responses

**Figure 21:**
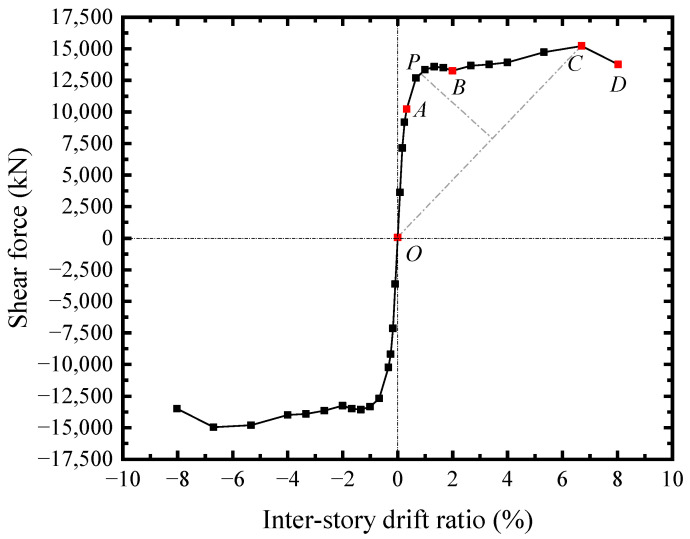
*V*-*θ* responses of the specimen TS10-B2032-C2048-TC150.

The shear force-drift ratio (*V*-*θ*) skeleton curve of TS10-B2032-C2048-TC150 was illustrated in Figure 21. The curve was basically linear until the infill steel web yielding at *θ* = 0.25% (point A). When the drift ratio ranged from 0.25% (point A) to 2.0% (point B), the SF-CPSW was into the elastoplastic phase. The elastoplastic phase of SF-CPSW was relatively long. It developed by the yielding of boundary beam webs, the concrete encasements reached the maximum shear force, and the yielding of columns webs successively. When drift ratio arrived at 0.66%, the shear force of concrete encasements reached the maximum, leading to *V* of SF-CPSW slightly descended until point B. The point P at *θ* = 0.84% was identified as the structural yield point of SF-CPSW following the “farthest point” rule, which has been presented in the test result. Beyond the point B, SF-CPSW entered the plastic phase, and the flanges of the beams yielded under the bending moments of the cross-section. At the drift ratio of 3.33%, the plastic buckling occurred in the steel web. However, the curve *V*-*θ* gradually ascended, since the boundary columns and beams were into the hardening range and capable of providing the load-carrying capacity. Because the contribution of boundary SF and the buckling restraints from concrete encasements, the buckling of the infill steel web and the deterioration of the concrete encasement had little impact on the overall shear carrying capacity, which ensured the ductility of SF-CPSW in the plastic phase. At *θ* = 6.67% (point C), *V* reached the peak of 15,196.7 kN. Subsequently, the flange of the boundary column buckled, and *V* dropped to 80% of its peak at the drift ratio of 8.0% (point D).

### 6.3. Responses of Infill Steel Web

**Figure 22:**
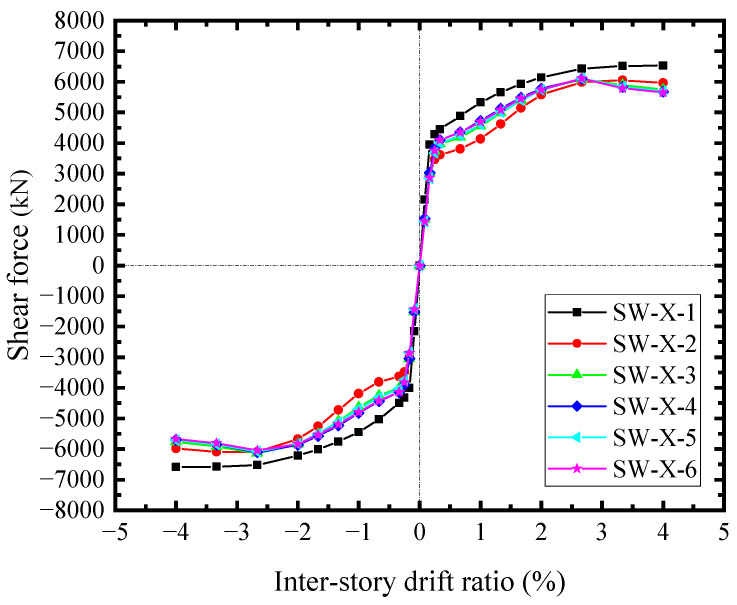
Horizontal shear forces of infill steel web.

The infill steel web shear forces along the horizontal and vertical directions were approximate. Figure 22 illustrated the horizontal counterparts. The shear forces at the edges of steel web (SW-X-1) were higher than the other sections, and it reached the shear yield force of 4291.7 kN at *θ* = 0.25%. The shear forces within the studs group (SW-X-3~SW-X-6) were close, and the outermost ones (SW-X-2) were the lowest. At *θ* = 3.33%, these shear forces significantly decreased due to the infill steel web buckling. The edge shear forces did not change, apparently. The vertical shear forces were similar to the horizontal counterparts, indicating that the main stress of infill steel web was the shear-dominated. In addition to the shear stress, some normal stresses were generated at the corner due to the interactions between the steel web and boundary SF, which affected the stress distribution of the steel web.

**Figure 23:**
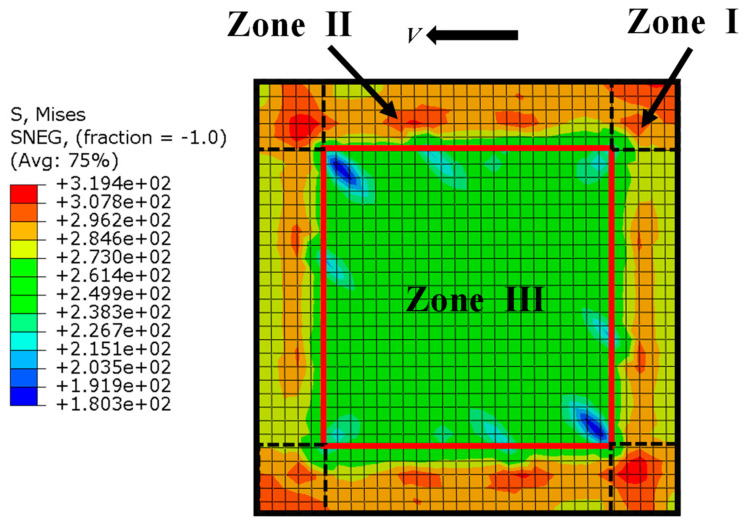
The Equivalent stress distribution of infill steel web.

Therefore, according to the stress distribution characteristics, the infill steel web was divided into three zones, as shown in Figure 23. Zone I was located at the corners of the infill steel web. Besides the remarkable shear stresses, there were some normal stresses resulting from the bending at the beam-column joints. These normal stresses affected the fully shear strength of steel web. Zone II and Zone III were mainly dominated by the shear stresses, where the shear stresses at the edge (Zone II) were higher than that in the middle (Zone III), because the infill steel web transferring part of the shear forces to the concrete encasements through the studs group corresponded to the location of Zone III. Since the outmost studs undertook the most part of shear transfers, the steel web cross-sections on these locations possessed the lowest shear forces, seeing the curve SW-X-2 in Figure 22. Overall, the deformation pattern of steel web was shear-dominated and considered to be mainly subjected in shear. Its shear forces were uniformly distributed and of a stable shear yield strength.

### 6.4. Responses of Concrete Encasements

**Figure 24:**
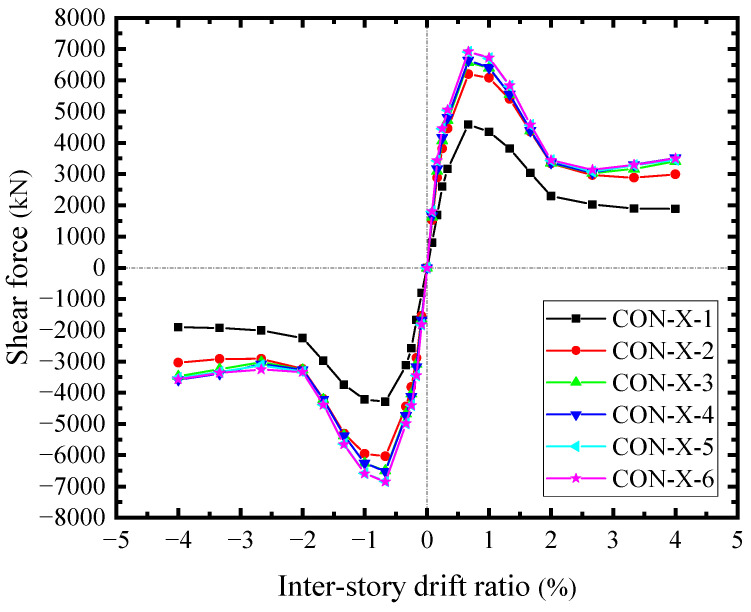
Horizontal shear forces of concrete encasements.

**Figure 25:**
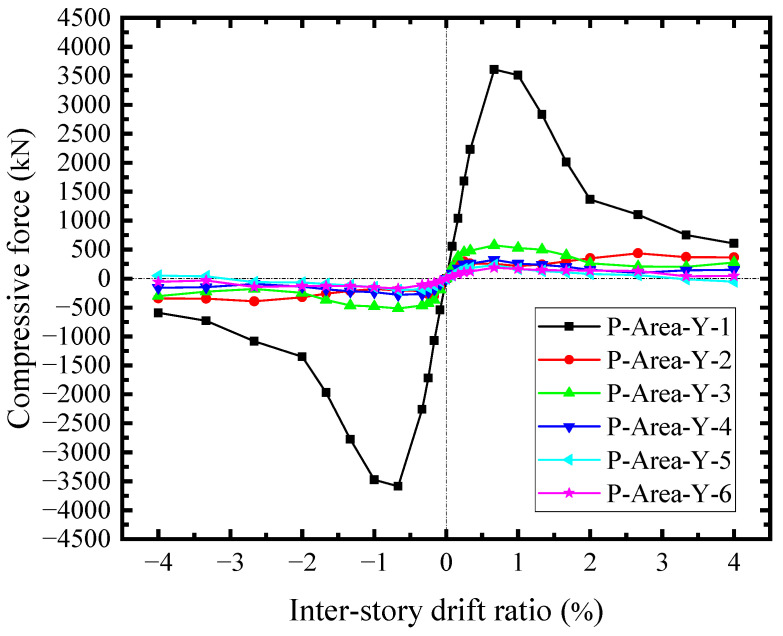
Vertical compressive forces of concrete encasements.

As the concrete encasements carried the shear forces from the steel web, the skeleton curves of the shear force-drift ratio across the horizontal sections were illustrated in Figure 24. The shear forces in the middle were approximate. The edge shear force was relatively low (CON-X-1). The edge shear forces were mainly transmitted by the compressions at the joints of boundary SF, while the shear forces in the middle had the additional shear forces transferred from the studs group. As a result, the concrete encasements significantly contributed to the shear resisting of SF-CPSW relying on the diagonal compression fields, which resulted from the shear of studs group and the compression at the joints of boundary SF. The difference between CON-X-1 and CON-X-2 reflected the shear force transferred by the studs group. In addition, these compressions constrained the bending deformation of SF, conversely, forming the same effects as the tensions at the corner of infill steel web (Zone I). Figure 25 illustrated the edge compressive force in different areas of concrete encasements along the vertical direction. The corner compressive force of the beam (P-Area-Y-1) was the largest and reached the maximum at *θ* = 0.66%, accounting for about 72.6% of the concrete encasement shear force at the edge (CON-X-1). And their development patterns were also similar. However, the edge compressive forces in the middle were negligible. The horizontal counterparts possess the same distribution and amplitude, implying that the interactions between CPSW and SF mainly focused on the column-beam joints.

### 6.5. Response of Boundary Columns and Beams

**Figure 26:**
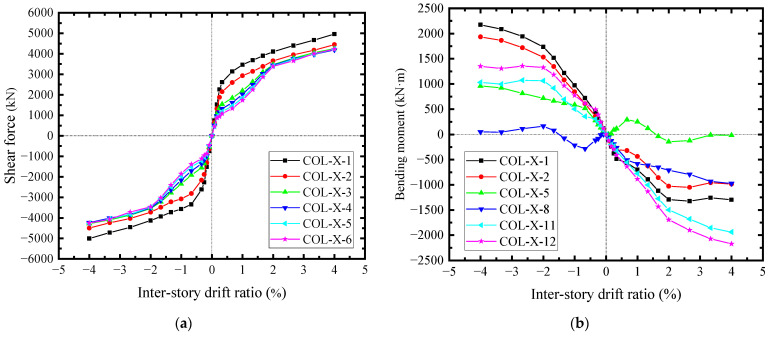
Shear forces and bending moments in the cross sections of the column. (**a**) Shear force. (**b**) Bending moment.

**Figure 27:**
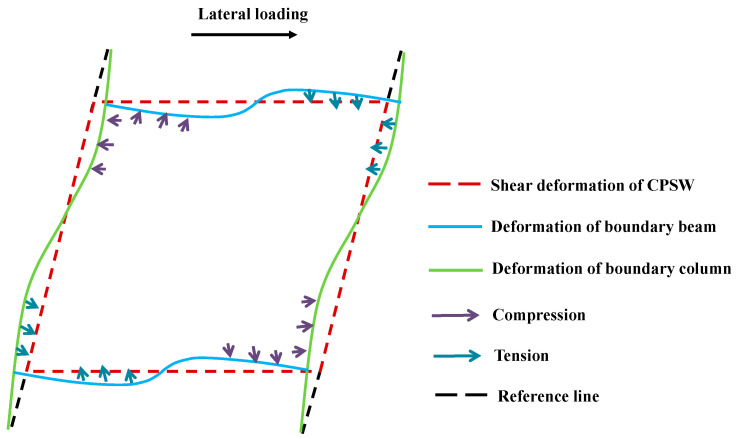
Deformation pattern of SF-CPSW.

The responses of the steel web and concrete encasements reflected the interactions between CPSW and boundary SF, whereby the internal forces of the boundary SF were affected. The shear forces of boundary columns were not relatively well-distributed before *θ* = 0.25%, as shown in Figure 26a. The middle shear forces (COL-X-3 ~ COL-X-6) were lower than the end ones (COL-X-1 and COL-X-2). The tensions from steel web corners (Zone I in Figure 23) and the compressions from concrete encasement corners (P-Area-Y-1 in Figure 25) confined the shear force development of columns and superimposed from the ends to the middle, which gradually decreased the sectional shear forces. After the severe deterioration of the concrete encasement shear capacity (*θ* > 2.0%), the column shear forces became close, due to the constraints from CPSW being significantly reduced. For the bending moments of the boundary column, these constraints from CPSW were more apparent, and some differences between the infill steel web and the concrete encasements were recognized, as shown in Figure 26b. The bending moments were asymmetrical under the positive and negative lateral loadings but were antisymmetric from both ends to the centerline of the column. For example, COL-X-1 along the negative direction was antisymmetric with COL-X-12 along the positive direction, because their correlative constraint was both induced by the compressions from concrete encasements. For the antisymmetric curves of COL-X-1 along positive direction and COL-X-12 along negative direction, the constraint was the tensions from steel web. It also implied that the constraint extent of the infill steel web was less than that of the concrete encasements. The interactions and acting forces under the relative deformations of CPSW and SF were depicted in Figure 27.

**Figure 28:**
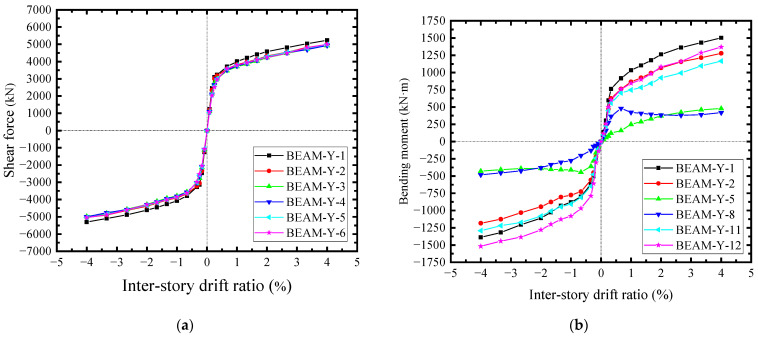
Shear forces and bending moments in cross sections of the beam. (**a**) Shear force. (**b**) Bending moment.

For the boundary beams, their acting forces were a little different, because they were subjected to the constraints from CPSWs in both the upper and lower storeys. As a result, the correlative constraints were stronger than that of the column, leading to the shear force distribution of beam being relatively uniform, as shown in Figure 28a. Accordingly, the beam-bending moments underwent the same acting forces from CPSW and also exhibited the similar development pattern as that of the columns, as shown in Figure 28b. In general, due to the constraints of CPSW, the bending deformations of the boundary SF were confined, whereby the yielding of boundary SF were postponed and started with the shearing. The shear yields of beam and column occurred at *θ* = 0.33% and 0.66%, respectively, seeing all curves in Figure 28a and curve COL-X-1in Figure 26a. After that, at *θ* = 1.66%, the flexural yields took place at the ends of beam and could not reach the relevant flexural strengths, attributing to the web shear yielding at the earlier stage, seeing curves BEAM-Y-1 and -12 in Figure 28b. For the columns, their end bending moment could not reach the theoretically flexural strength, either, seeing the reflecting points at *θ* = ±3.33% of the curves COL-X-1 and -12 in Figure 26b. Consequently, the lateral resistance of boundary SF depends on the shear strength of columns and the flexural capacity of the column-beam joint, guaranteeing the ductility of SF-CPSW.

### 6.6. The Shear Stiffness and Resistance Assignments of Structural Elements

**Figure 29:**
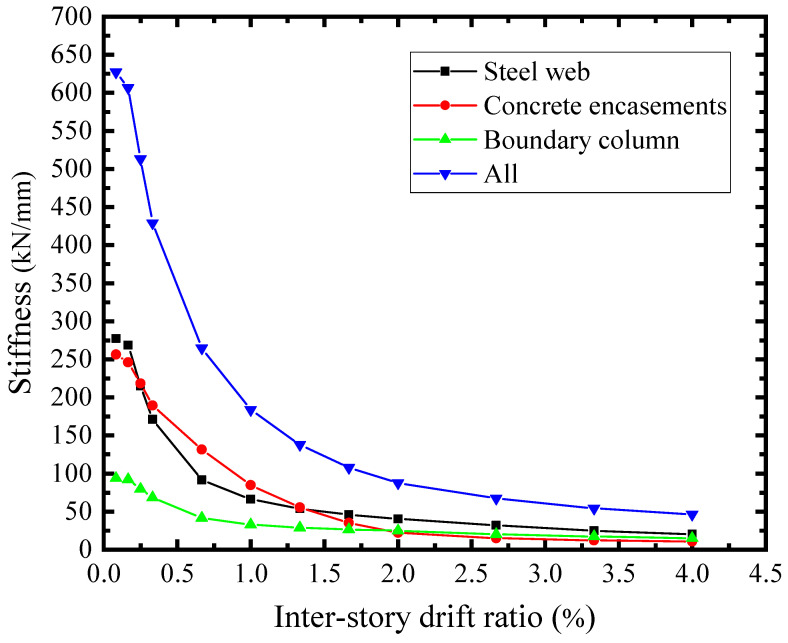
Stiffness degradation.

**Figure 30:**
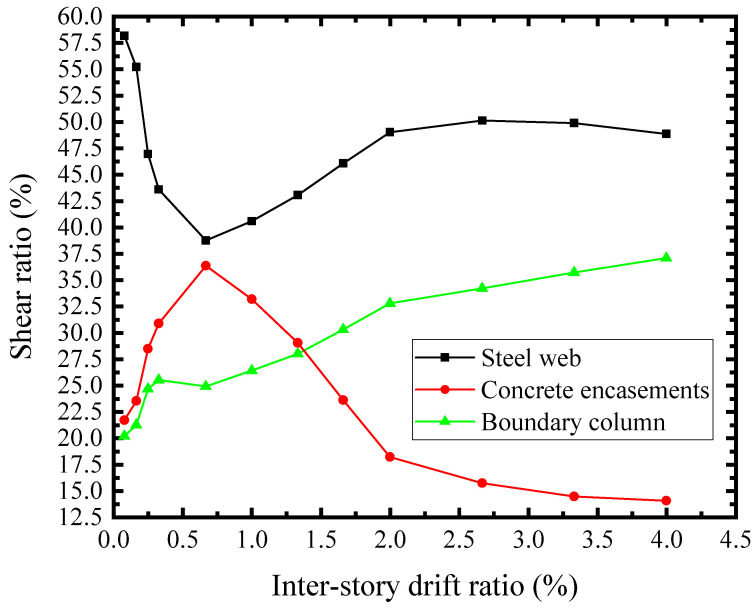
Shear force assignment.

The lateral load of SF-CPSW was carried by the infill steel web, concrete encasements, and boundary columns. The shear stiffness-drift ratio relationships of the SF-CPSW and the structural elements were presented in Figure 29. The initial stiffness of the infill steel web and the concrete encasements were 277 kN/mm and 256 kN/mm, respectively. Their combination accounted for 84.7% of the total stiffness, while the contribution of boundary column was relatively low. As shown in Figure 30, their shear force proportion of the total shear resistance further proved this. The infill steel web and concrete encasements were the primary shear resisting structural elements before *θ* = 0.66%, corresponding to the *V* of concrete encasements reaching the maximum. After the shear yielding of the infill steel web (*θ* = 0.25%), the shear force proportion of the columns increased rapidly. Ultimately, the proportion of the columns exceeded that of the concrete encasements. From the above analysis, it could be concluded that the shear performance of SF-CPSW was dominated by the infill steel web. The concrete encasements participated in the shear resistance at the early phase, which improved the shear stiffness and yield strength of SF-CPSW. The columns played a role of “back-up system” to ensure the ductility of the SF-CPSW.

## 7. Parametric Analysis

**Figure 31:**
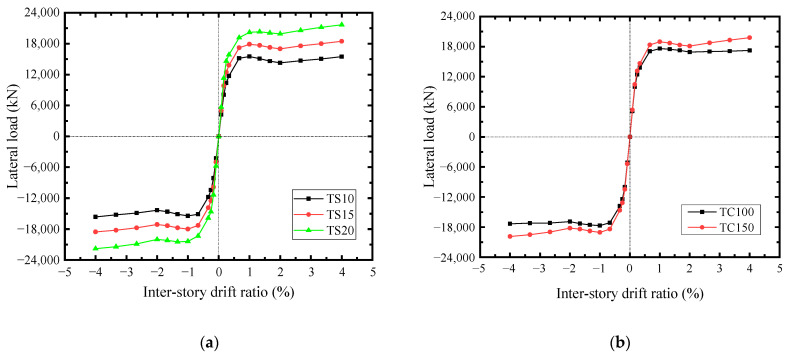

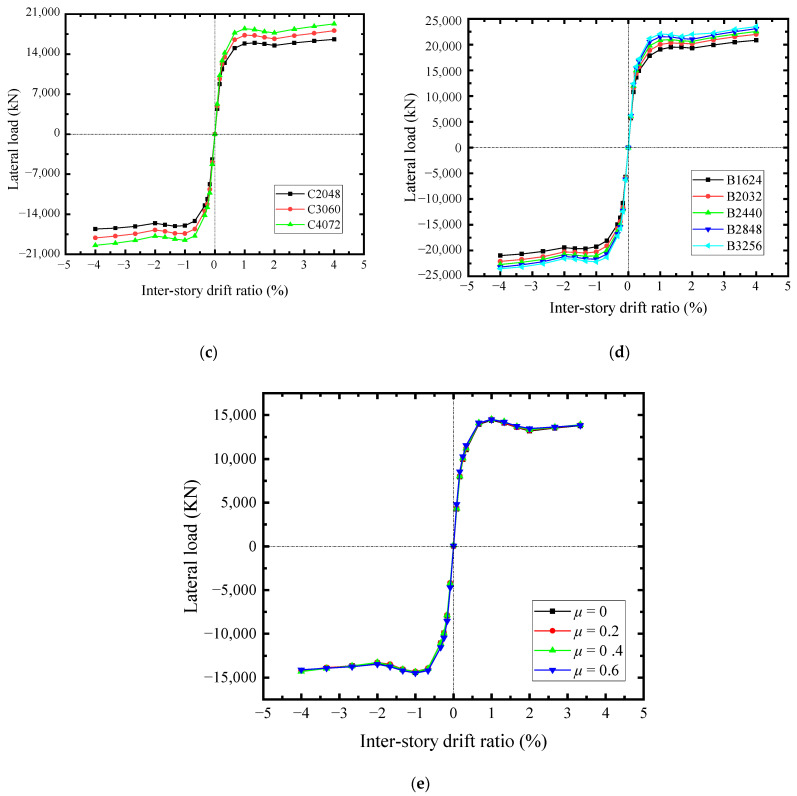
*V*-*θ* skeleton curves of FEM specimens with different parameters. (**a**) Steel web thicknesses. (**b**) Concrete encasements thicknesses. (**c**) Column cross-sections. (**d**) Beam cross-sections. (**e**) Axial compression ratios.

Based on the behaviors and interactions of structural elements, the effects of infill steel web and concrete encasements thicknesses, the cross-sections of boundary SF, and the axial compression ratio on the lateral-load resistance of SF-CPSW were analyzed. The varying parameters used were shown in Table 2. The infill steel web was the major member for the lateral resistance of SF-CPSW. As shown in Figure 31a, “TS-” denoted the infill steel web thickness. With the thickness of infill steel web increased 5 mm, the shear yield force of the SF-CPSW increased by about 23.5%. When the concrete encasement thickness (TC-) increased, the shear stiffness and yield force of SF-CPSW improved accordingly, and the buckling of the infill steel web delayed, which improving the ductility of SF-CPSW, as shown in Figure 31b. For the boundary SF, enlarging the cross-section of column (C-) enhanced the lateral-load resistance especially after initial yielding of SF-CPSW, as shown in Figure 31c. The beam cross-section (B-) also affected the lateral-load capacity of SF-CPSW, because it should have the capacities to transfer the vertical shear from the infill steel web to ensure the sufficient flexural capacity of the beam-to-column joints, so that decreasing the beam cross-section reduced the lateral resistance of SF-CPSW, shown in Figure 31d. When the boundary SF was adequate to the vertical load, the axial compression ratio (μ) of columns slightly affected the lateral-load capacity of SF-CPSW, as shown in Figure 31e.

## 8. Simplified *V*-*θ* Model and Formulas

**Figure 32:**
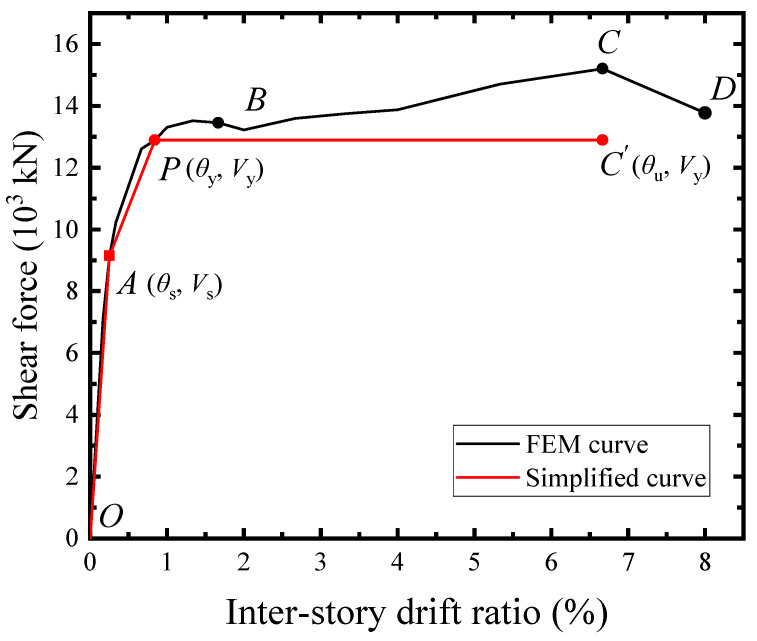
*V*-*θ* relationship of simplified model.

To provide the shear stiffness and strengths of SF-CPSW for the seismic design or elastoplastic analysis of the structure, the simplified mechanical model was proposed for the *V*-*θ* response, as shown in Figure 32. The simplified model incorporating the elastic phase (OA), the elastoplastic phase (AP), and the plastic phase (PC’) was used to fit the *V*-*θ* curve O-A-B-C-D. O-A-P-C’ could accurately capture the changing characteristics of O-A-B-C-D and correspond to the mechanical characteristics of the structural elements with a sufficient safety margin and reasonably predict.

The point A was the initial yield point of SF-CPSW, corresponding to the yield onset of infill steel web. Its shear force *V*_s_ and secant slope representing the initial stiffness *K*_s_ of SF-CPSW were both provided from the infill steel web, concrete encasements, and boundary column. As expressed in Equations (1) and (2), *f*_ys_ was the yield strength of the infill steel web in MPa; *f*_c_ was the nominal compressive strength of concrete in MPa; *f*_yc_ was the yield strength of the columns; *t*_s_, *t*_c_ and *t*_cw_ were the infill steel web, concrete encasement, and column web thicknesses, respectively; *l* was the width of CPSW, which was the same for the infill steel web and concrete encasements; *h*_c_ was the height of the column web; and *G*_s_ and *G*_c_ were the shear modulus of steel and concrete, respectively.

The infill steel web was mainly in shear, and its contribution was given by 0.58 *f*_ys_*t*_s_*l*. The concrete encasements carried the shear force through the diagonal compression fields, where their shear capacity was given as 0.12 *f*_c_*t*_c_*l* at point A. For the columns, their mechanical characteristic was dominated by shear under the constraints from CPSW, while the webs did not yield at this point. Such that, its contribution to Vs was calculated by a reduction shear yield strength, considered as 0.55 × 0.58 *f*_yc_*t*_cw_*h*_c_ = 0.32 *f*_yc_*t*_cw_*h*_c_. As a result, the formulas of the initial yield force *V*_s_ and the initial stiffness *K*_s_ of SF-CPSW were established.

(1) $$V_{s}=0.58f_{ys}t_{s}l+0.12f_{c}t_{c}l+0.32f_{yc}t_{cw}h_{c}$$

(2) $$K_{s}=0.72G_{s}t_{s}l+0.15G_{c}t_{c}l+0.41G_{s}t_{cw}h_{c}$$

The point P represented the structural yielding of SF-CPSW, identified by the “farthest point” rule, where the concrete encasement shear force just passed through the peak point with insignificantly dropping, and the shear yielding sequentially occurred in the boundary beams and column. Compared with point A, the contribution of concrete encasements increased, while the contribution of infill steel web slightly decreased due to its fully shear yielding. Meanwhile, the reduced constraints of CPSW caused the increasing of bending deformations at the column-beam joints. Consequently, the contribution of columns to the load-carrying additionally relied on the flexural capacity of joints. Equations (3) and (4) expressed the shear yield force *V*_y_ and the correlative stiffness *K*_y_ as the analysis above, where *I*_b_ and *I*_c_ were the sectional moment of inertias of boundary beam and column, respectively.

(3) $$V_{y}=0.77f_{ys}t_{s}l+0.16f_{c}t_{c}l+0.745{\left(\frac{I_{b}}{I_{c}}\right)}^{0.524}f_{yc}t_{cw}h_{c}$$

(4) $$K_{y}=0.083G_{s}t_{s}l+0.06G_{c}t_{c}l+0.108{\left(\frac{I_{b}}{I_{c}}\right)}^{0.266}G_{s}t_{cw}h_{c}$$

**Figure 33:**
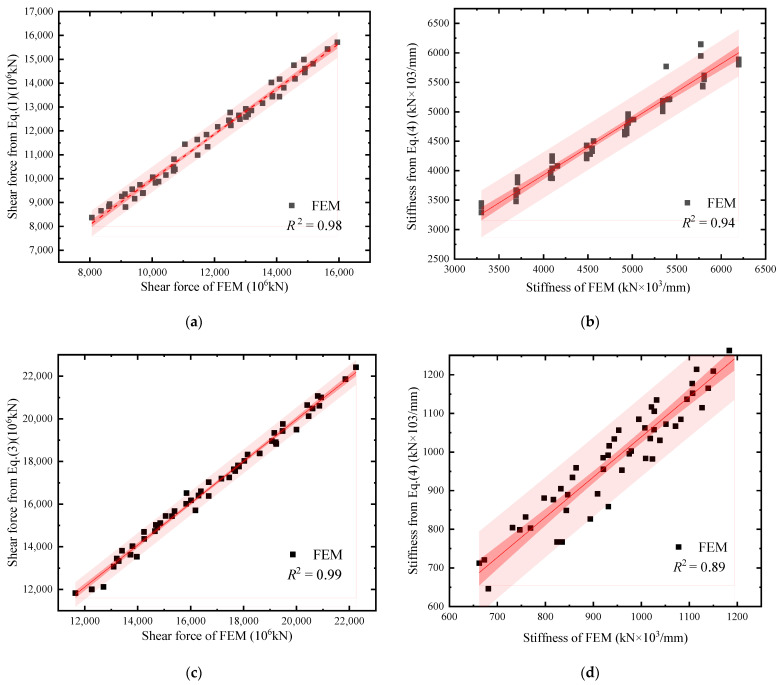
Comparison in strengths and stiffness. (**a**) Initial yield force *V*_s_. (**b**) Initial stiffness *K*_s_. (**c**) Shear yield force *V*_y_. (**d**) Shear stiffness *K*_y_.

Considering the structural safety, the formulas fitting of *V*_s_, *K*_s_, *V*_y_, and *K*_y_ tended to be conservative. The relevant fitting data were provided in Table A1 and Table A2 of Appendix A. The errors of *V*_s_, *K*_s_, *V*_y_, and *K*_y_ between FEM and the proposed formulas were analyzed by variances and confidence intervals, as shown in Figure 33. It was demonstrated that the proposed formulas and simplified *V*-*θ* model can be effectively used in the seismic design of SF-CPSW with typical dimensions and constructions.

## 9. Discussion

In this paper, the lateral-load resisting mechanism of steel frame infilled with composite plate shear wall (SF-CPSW) was revealed based on the interactions of structural members. The prior investigations predominantly focused on the shear capacity of CPSW and the overall lateral-load resistance of SF-CPSW [1,2,3,4,5,6,11,12,13,14,15,16,17,18], which demonstrated the excellent performance of SF-CPSW under cyclic loading, and explored the constructions, shapes, and materials for improvement. The lateral load assignment between SF and CPSW was studied by comparing the lateral resistances between individual SF and SF-CPSW [22]. Notably, limited attention was paid on the interactions between boundary SF and CPSW. The normal stresses were considered to be the major ones resulting from the interactions between SF and CPSW and linearly distributed along the edges of steel web [24], which neglected the bending deformation of SF and the influences from concrete encasements. In this paper, such interactions were found to originate from the bending constraints of CPSW to SF. The corresponding acting forces affected the mechanics characteristics of infill steel web and concrete encasements, where the normal stresses at the corner were relatively high, reducing the shear strength of steel web. Attributing to the load-resisting contributions from concrete encasements and boundary SF, however, the steel web could reach the theoretical value of fully shear yield strength. Furthermore, such contributions improved the stiffness and ductility of SF-CPSW.

Based on the analysis results, the simplified shear force-drift ratio model with formulas of shear strengths and stiffness were proposed. For the seismic design, these formulas could be applied to the SF-CPSW with typical dimensions and constructions, including the wall aspect ratio of 1.0, the height-thickness ratio of steel web ranging from 150 to 300, and the concrete encasements attached to both sides of steel web. The further researching directions should be on the shape, the aspect ratio, and the constructions of the filling shear wall to provide more technical supports on the applications of SF-CPSW.

## 10. Conclusions

To reveal the mechanism of lateral-load resistance in steel frame infilled with composite plate shear wall (SF-CPSW), this paper studied the lateral resistance, failure mode, internal forces, and interactions of SF-CPSW by a structural model test and finite element analyses, based on which the simplified shear force-drift ratio model with formulas of shear strengths and stiffness were proposed for the seismic design. The conclusions were summarized in the following points:

(1) SF-CPSW performs considerable lateral resistance and ductility as the coordinated deformations between SF and CPSW under cyclic loading. CPSW dominates the major shear behaviors with its infill steel web, and confines the bending of SF, thereby ensuring the “back-up” system.

(2) The elastoplastic phase of SF-CPSW is relatively long, where it commences with the initially shear yielding of infill steel web and develops by the yielding of boundary beams, the concrete encasements reaching the maximum shear force, and the yielding of columns successively.

(3) The corners of infill steel web featured by the high tensile stresses to constrain the bending of SF, and its panel with the stud groups, possess a lower shear stress level than that of the edges. However, steel web shear forces are uniform distributed and of a stable shear yield strength because the deformation pattern of SF-CPSW is shear dominated.

(4) The concrete encasements significantly contribute to the shear stiffness and yield strength of SF-CPSW relying on the diagonal compression fields. Furthermore, they constrain the bending deformation of SF and postpone the plastic buckling of infill steel web, improving the ductility of SF-CPSW.

(5) The lateral resistance of boundary columns depends on its shear strength and the flexural capacity of the column-beam joint, and they guarantee the ductility of the SF-CPSW.

## Figures and Tables

**Table 1:** Material properties of different steel web thickness.

*t*_0_ (mm)	*f*_y_ (N/mm^2^)	*f*_u_ (N/mm^2^)	*E*_s_ (N/mm^2^)	*δ*
3	313	428	202.60	25%
5	280	413	196.57	29%
8	249	398	173.92	33%
10	252	409	185.77	34%
16	254	421	162.74	33%

**Table 2:** Parameters of SF-CPSW.

Specimen	Steel Web Thickness (mm)	Beam Section (mm)	Column Section (mm)	Concrete Thickness (mm)	Axial Compression Ratio
TSx-Bx-Cx-TCx-x	10	20 × 32	30 × 60	150	0.2, 0.4, 0.6
	10	16 × 24, 20 × 32, 24 × 40, 28 × 48, 32 × 56	20 × 48, 30 × 60, 40 × 72	100, 150	0
	12.5				
	15				
	17.5				
	20				

## Data Availability

The data that support the findings of this study are available from the corresponding author upon reasonable request. The data are not publicly available due to privacy.

## Appendix A

**Table A1:** The horizontal shear forces of each component at initial yielding (at Point A).

Specimens	Steel Web	Concrete Encasements	Boundary Column	Combined Shear Force	Equation (1)
TS10-B1624-C2048-TC150	4294.1	2100.6	2408.7	8803.4	9144.5
TS10-B1624-C2048-TC100	4316.2	2097.1	1953.9	8367.2	8064.5
TS10-B2032-C2048-TC150	4291.8	2261.2	2601.5	9154.5	9439.6
TS10-B2032-C2048-TC100	4313.4	2261.4	2074.5	8649.3	8359.6
TS10-B2440-C2048-TC150	4292.4	2368.1	2720.9	9381.3	9693.9
TS10-B2440-C2048-TC100	4313.3	2364.3	2155.8	8833.4	8613.9
TS10-B1624-C3060-TC150	4336.8	2397.4	2668.0	9402.3	9711.7
TS10-B1624-C3060-TC100	4353.8	2406.7	2167.3	8927.7	8631.7
TS10-B2032-C3060-TC150	4334.7	2607.5	2881.5	9823.7	10,103.7
TS10-B2032-C3060-TC100	4316.2	2624.5	2309.3	9250.0	9023.7
TS10-B2440-C3060-TC150	4330.7	2782.7	3036.2	10,149.6	10,441.4
TS10-B2440-C3060-TC100	4350.4	2791.8	2412.8	9555.1	9361.4
TS10-B2848-C3060-TC150	4332.7	2905.3	3145.6	10,383.6	10,736.8
TS10-B1624-C4072-TC150	4365.4	2648.8	2858.2	9872.3	10,211.3
TS10-B1624-C4072-TC100	4379.0	2666.1	2310.0	9355.1	9131.3
TS10-B2032-C4072-TC150	4361.5	2884.1	3078.2	10,323.8	10,688.6
TS10-B2032-C4072-TC100	4384.1	2902.6	2453.2	9739.9	9608.6
TS10-B2440-C4072-TC150	4140.2	3092.1	3246.6	10,478.8	10,688.6
TS10-B2440-C4072-TC100	4378.3	3112.0	2568.4	10,058.7	10,019.8
TS10-B2848-C4072-TC150	4358.1	3266.9	3357.3	10,982.3	11,459.5
TS12.5-B2848-C3060-TC150	5385.1	2717.3	3333.9	11,436.5	11,052.7
TS12.5-B2032-C4072-TC150	5411.3	2670.0	3291.9	11,373.3	12,507.1
TS12.5-B2440-C4072-TC150	5411.3	2808.2	3549.7	11,769.4	12,507.1
TS12.5-B2848-C4072-TC150	5413.6	2897.0	3765.2	12,076.0	13,015.7
TS15-B2440-C2048-TC150	6239.7	2088.3	2720.9	11,330.3	11,781.9
TS15-B2440-C2048-TC100	6323.4	2107.7	1590.4	10,808.0	10,701.9
TS15-B2440-C3060-TC150	6331.4	2318.4	2337.9	12,229.9	12,529.4
TS15-B2440-C3060-TC100	6397.2	2382.9	1819.6	11,636.8	11,449.4
TS15-B2848-C3060-TC150	6368.7	2515.9	2445.6	12,486.8	12,824.8
TS15-B2848-C3060-TC100	6398.4	2507.2	1902.4	11,839.2	11,744.8
TS15-B3256-C3060-TC150	6415.6	2471.4	1893.5	12,685.9	13,085.4
TS15-B2440-C4072-TC150	6441.3	2770.7	2606.2	12,845.0	13,187.8
TS15-B2440-C4072-TC100	6477.1	2775.6	2059.4	12,169.0	12,107.8
TS15-B2848-C4072-TC150	6456.4	2994.2	2779.4	13,158.0	13,547.5
TS15-B2848-C4072-TC100	6483.8	2982.7	2170.3	12,443.9	12,467.5
TS15-B3256-C4072-TC150	6458.3	3145.9	2882.6	13,426.5	13,864.8
TS15-B3256-C4072-TC100	6483.7	3121.7	2233.8	12,646.5	12,784.8
TS17.5-B2848-C3060-TC150	6455.4	3257.0	2973.5	13,465.1	13,868.8
TS17.5-B2440-C4072-TC150	6490.5	3068.0	2818.6	13,804.1	14,231.8
TS17.5-B2848-C4072-TC150	6515.9	3072.0	2212.0	14,177.9	14,591.5
TS17.5-B3256-C4072-TC150	6506.8	3335.1	3003.1	14,443.0	14,908.8
TS20-B2848-C2048-TC150	6528.5	3314.6	2325.9	13,430.1	14,092.3
TS20-B2848-C2048-TC100	6506.8	3527.3	3124.0	12,921.2	13,012.3
TS20-B2848-C3060-TC150	6529.2	3506.0	2408.7	14,605.0	14,912.8
TS20-B2848-C3060-TC100	6501.9	3693.2	3231.5	14,021.7	13,832.8
TS20-B3256-C3060-TC150	6528.8	3649.1	2468.6	14,809.5	15,173.4
TS20-B3256-C3060-TC100	7482.2	2514.2	3468.6	14,168.4	14,093.4
TS20-B2848-C4072-TC150	7456.5	2445.6	3355.1	15,424.3	15,635.5
TS20-B2848-C4072-TC100	7521.8	2608.9	3673.3	14,750.1	14,555.5
TS20-B3256-C4072-TC150	7537.7	2722.6	3917.5	15,711.3	15,952.8
TS20-B3256-C4072-TC100	7539.0	2798.0	4105.9	14,982.7	14,872.8

**Table A2:** The horizontal shear forces of each component at the structural yielding (at Point P).

Specimens	Steel Web	Concrete Encasements	Boundary Column	Combined Shear Force	Equation (3)
TS10-B1624-C2048-TC150	5115.2	3833.2	3166.0	12,114.4	12,711.9
TS10-B1624-C2048-TC100	5188.9	3691.4	3119.4	11,999.7	11,267.4
TS10-B2032-C2048-TC150	5224.1	4462.5	3380.4	13,066.9	13,093.7
TS10-B2032-C2048-TC100	5223.2	4208.2	3392.6	12,824.0	11,649.2
TS10-B2440-C2048-TC150	5228.5	5055.4	3526.5	13,810.4	13,413.9
TS10-B2440-C2048-TC100	5232.0	4808.4	3486.6	13,527.0	11,969.4
TS10-B1624-C3060-TC150	5220.0	4590.0	3808.5	13,618.5	13,732.6
TS10-B1624-C3060-TC100	5236.3	4379.8	3712.4	13,328.4	12,288.1
TS10-B2032-C3060-TC150	5253.5	5039.0	4078.4	14,370.9	14,252.1
TS10-B2032-C3060-TC100	5188.9	4840.9	3992.7	14,022.6	12,807.6
TS10-B2440-C3060-TC150	5261.8	5400.0	4359.7	15,021.5	14,687.7
TS10-B2440-C3060-TC100	5262.6	5180.6	4254.0	14,697.1	13,243.2
TS10-B2848-C3060-TC150	5252.8	5691.3	4495.7	15,439.8	15,060.8
TS10-B1624-C4072-TC150	5280.8	5038.8	4411.8	14,731.4	14,655.8
TS10-B1624-C4072-TC100	5289.5	4875.1	4282.6	14,447.2	13,211.3
TS10-B2032-C4072-TC150	5317.4	5451.9	4667.8	15,437.1	15,299.7
TS10-B2032-C4072-TC100	5318.6	5243.2	4549.0	15,110.7	13,855.2
TS10-B2440-C4072-TC150	5317.4	5747.1	4947.8	16,012.4	15,839.8
TS10-B2440-C4072-TC100	5324.2	5501.0	4849.1	15,674.3	14,395.3
TS10-B2848-C4072-TC150	5318.8	5934.7	5147.0	16,400.5	16,302.3
TS12.5-B2848-C3060-TC150	6323.2	5038.0	5153.0	16,514.1	14,848.7
TS12.5-B2032-C4072-TC150	6353.6	4654.0	5373.8	16,381.3	16,686.5
TS12.5-B2440-C4072-TC150	6384.9	4978.8	5658.4	17,022.0	16,686.5
TS12.5-B2848-C4072-TC150	6396.3	5190.3	5957.0	17,543.6	17,689.0
TS15-B2440-C2048-TC150	7626.9	4597.0	3485.1	15,709.0	16,187.3
TS15-B2440-C2048-TC100	7652.4	3751.1	3499.2	14,902.7	14,742.8
TS15-B2440-C3060-TC150	7704.7	5130.0	4409.9	17,244.6	17,461.2
TS15-B2440-C3060-TC100	7748.9	4033.9	4390.1	16,172.8	16,016.7
TS15-B2848-C3060-TC150	7704.7	5400.0	4660.4	17,765.1	17,834.3
TS15-B2848-C3060-TC100	7761.2	4168.6	4673.4	16,603.1	16,389.8
TS15-B3256-C3060-TC150	7732.6	5712.1	4878.7	18,323.4	18,158.1
TS15-B2440-C4072-TC150	7815.6	5509.9	5050.5	18,376.0	18,613.2
TS15-B2440-C4072-TC100	7856.1	4254.4	5082.8	17,193.3	17,168.7
TS15-B2848-C4072-TC150	7835.2	5785.0	5333.6	18,953.8	19,075.8
TS15-B2848-C4072-TC100	7870.0	4353.0	5403.4	17,626.5	17,631.3
TS15-B3256-C4072-TC150	7833.9	5961.7	5634.4	19,430.0	19,477.1
TS15-B3256-C4072-TC100	7871.0	4417.2	5730.5	18,018.7	18,032.6
TS17.5-B2848-C3060-TC150	8953.3	4659.8	5293.3	18,906.3	19,221.0
TS17.5-B2440-C4072-TC150	9041.6	4577.6	5870.2	19,489.4	19,999.9
TS17.5-B2848-C4072-TC150	9089.1	4812.3	6218.7	20,120.0	20,462.5
TS17.5-B3256-C4072-TC150	9099.5	4943.7	6565.9	20,609.2	20,863.9
TS20-B2848-C2048-TC150	10,189.4	4860.0	3775.1	18,824.5	19,235.0
TS20-B2848-C2048-TC100	10,217.8	3892.5	3702.7	17,813.0	17,790.5
TS20-B2848-C3060-TC150	10,440.0	5130.0	4911.0	20,481.0	20,607.7
TS20-B2848-C3060-TC100	10,438.5	4176.4	4727.4	19,342.3	19,163.2
TS20-B3256-C3060-TC150	10,440.0	5400.0	5161.5	21,001.5	20,931.5
TS20-B3256-C3060-TC100	10,474.2	4295.4	4987.5	19,757.2	19,487.0
TS20-B2848-C4072-TC150	10,629.8	5518.7	5712.2	21,860.7	21,849.2
TS20-B2848-C4072-TC100	10,638.6	4356.3	5649.0	20,643.9	20,404.7
TS20-B3256-C4072-TC150	10,678.0	5723.5	6017.0	22,418.4	22,250.6
TS20-B3256-C4072-TC100	10,660.0	4426.8	5981.7	21,068.5	20,806.1
